# Cases of Wounded Arteries of the Arm, Relieved by the Bandage

**Published:** 1841-07

**Authors:** George Thompson

**Affiliations:** Jefferson, Tennessee


ART. IV.—*Cases of Wounded Arteries of the Arm, relieved by the Bandage:* Reported to the Medical Society of Tennessee, May, 1841. By Dr. GEORGE THOMPSON, of Jefferson, Tenn.

*Case* 1.—A young man received a wound from a long knife in the fore-arm. The knife entered about the middle of the fore-arm, and, passing obliquely upward, wounded the ulna artery just below the point of separation from the radical. The hemorrhage had been considerable, but was arrested by the application of a bandage around the arm above the elbow. In this situation he came to my shop, an hour after he had received the injury. A compress was laid over the course of the wound, and another over the brachial artery, at the point where that vessel could be most conveniently compressed against the humerus. A roller was then carefully applied from the points of the fingers to the shoulder, so tightly as barely to permit a sufficient quantity of circulation to maintain the vitality of the limb. Cold water was freely applied to the whole arm; he was put on light diet, with an occasional dose of epsom salts. I removed and re-adjusted the bandage daily, to satisfy myself that the limb was receiving no injury from it. In ten or twelve days the wound was healed. On removing the bandage the circulation was found to be carried on through the wounded vessel near the wrist as freely as before the wound was received. I could not satisfy myself that the canal of the vessel was not oblitered at the wounded point, from the depth with which it was covered by the integuments; but I am of the opinion it was not.

*Case* 2.—A young man passed a sharp pointed narrow knife under the tendon of the extensor muscle of the thumb, entering at the point where the radical artery passes under that tendon, wounding that vessel and passing out at the opposite side of the wrist. A compress was applied along the course of the wound, another on the vessel above the wound, a bandage was firmly applied to the hand and fore-arm, and the patient was left with instruction to let me know if the bandage produced much pain. His hand becoming painful in the night, he had the bandage taken off and applied loosely. I heard nothing more from him for four or five days, when he came to me to examine his hand. On taking off the bandage, which was quite loose, I found the wounds in the skin healed, and a strongly pulsating tumor along the whole course of the wound. The compresses and bandage were again applied as at first. The bandage was now permitted to remain as I applied it, and in three weeks all traces of aneurism had disappeared, and the hand was soon restored to its original condition.

